# PReS-FINAL-1008: Areas of psychological assistance to children with severe disease during rehabilitation

**DOI:** 10.1186/1546-0096-11-S2-P6

**Published:** 2013-12-05

**Authors:** A Buslaeva

**Affiliations:** 1 Scientific Center of Children's Health RAMS, Moscow, Russian Federation

## Introduction

Now there is a significant increase in the number of children with rheumatic diseases. That in return raises the question of the necessity of studying the psychological difficulties that has a child suffering from the disease, as well as provide direction for psychological assistance.

## Objectives

41 children aged from 7 to 17 years with rheumatic diseases. The first group (grave disease) - 15 persons, the second group (mild illness) - 26 people. We examined emotional sphere, requirement-motivational sphere, features of autoassessment, - relationship with peers.

## Methods

Modified test "Painting of a man" (autopainting, a sick person and a healthy person painting), the method of "self-assessment" Dembo-Rubinshtey, the test "Three Wishes" and the method of "complete sentences".

## Results

Results of the study of children and adolescents with rheumatic diseases in remission, whose health was assessed by doctors as a condition of the medium severity, showed **t**hat their psychological characteristics are broadly consistent with age norms, and they quickly adapted to the conditions of the hospital. They noted emotional stability, good humor, a wide range of diverse desires specific to each age group. Self-evaluation of the majority of children surveyed were positive.

All children with severe rheumatic diseases were for a long time treated in hospital, their psychological state was directly dependent on the physical. Most vividly emotional difficulties were expressed in the form of reduced background mood, increased emotional lability, acute emotional reactions, in some cases accompanied by suicidal thoughts.

Children from both groups suffered staying at hospital and had un urgent need to unite with the family. Slightly less than half of the children in the first group on health could not leave the ward or get out of bed because of compression fracture of the spine, which greatly reduces the chances of a child to communicate with peers, satisfy his educational interests, carry out any activities. Enforced isolation had a negative impact on the interests of the child. The severity of the physical and psychological status had a negative impact on the image, self-esteem nature and ego.

## Conclusion

The direction of psychological help in the process of treatment must be tailored to the nature of the disease, the age and psychological characteristics of the child.

Children with severe disease require individual form of psychological help in the treatment process to reduce emotional stress, and with the wider community needs, switch the child's attention from the negative experiences to the joint activity with the specialist.

The work in group is good for children with the medium severity of disease. Through communication and collaboration it will learn them to get and to give the emotional support to their peers, to find the self-support ways, plan their own time, realize their own emotional states and needs and also enhance the self-respect and personal value sense. The group form work will expand the range of interests of the child, develop communication skills and self-presentation.

## Disclosure of interest

None declared.

